# The molecular basis for selective assembly of the UBAP1-containing endosome-specific ESCRT-I complex

**DOI:** 10.1242/jcs.140673

**Published:** 2014-02-01

**Authors:** Lydia Wunderley, Kim Brownhill, Flavia Stefani, Lydia Tabernero, Philip Woodman

**Affiliations:** Faculty of Life Sciences, University of Manchester, Manchester M13 9PT, UK

**Keywords:** EGFR, Endosome, TSG101, ESCRT-I, UBAP1, VPS37, MVB12

## Abstract

ESCRT-I is essential for the multivesicular body (MVB) sorting of ubiquitylated cargo such as epidermal growth factor receptor, as well as for several cellular functions, such as cell division and retroviral budding. ESCRT-I has four subunits; TSG101, VPS28, VPS37 and MVB12. There are several members of VPS37 and MVB12 families in mammalian cells, and their differential incorporation into ESCRT-I could provide function-specific variants of the complex. However, it remains unclear whether these different forms of VPS37 and MVB12 combine randomly or generate selective pairings within ESCRT-I, and what the mechanistic basis for such pairing would be. Here, we show that the incorporation of two MVB12 members, UBAP1 and MVB12A, into ESCRT-I is highly selective with respect to their VPS37 partners. We map the region mediating selective assembly of UBAP1–VPS37A to the core ESCRT-I-binding domain of VPS37A. In contrast, selective integration of UBAP1 requires both the minimal ESCRT-I-binding region and a neighbouring predicted helix. The biochemical specificity in ESCRT-I assembly is matched by functional specialisation as siRNA-mediated depletion of UBAP1, but not MVB12A and MVB12B, disrupts ubiquitin-dependent sorting at the MVB.

## INTRODUCTION

Activated epidermal growth factor receptor (EGFR) is ubiquitylated, then internalised and sorted to the multivesicular body (MVB) en route to its degradation in the lysosome (Eden et al., 2012; Sorkin and Goh, 2009). MVB sorting of ubiquitylated EGFR involves several ubiquitin-binding protein complexes, termed endosomal sorting complex required for transport (ESCRT)-0, -I and -II (Bishop et al., 2002; Doyotte et al., 2005; Garrus et al., 2001; Henne et al., 2011; Hurley and Stenmark, 2011; Komada and Soriano, 1999; Malerød et al., 2007; Raiborg and Stenmark, 2009; Raiborg et al., 2002; Razi and Futter, 2006; Ren and Hurley, 2010; Slagsvold et al., 2005; Wollert and Hurley, 2010). Although the function of ESCRT-II is less well characterised, ESCRT-0 and ESCRT-I combine with the scaffold protein, his domain protein tyrosine phosphatase (HD-PTP, also known as PTPN23) (Ali et al., 2013; Doyotte et al., 2008; Stefani et al., 2011) and the deubiquitylating enzyme, UBPY/USP8 (Alwan and van Leeuwen, 2007; Bowers et al., 2006; Pareja et al., 2012; Row et al., 2006), to facilitate the transfer of EGFR to a further complex, ESCRT-III. ESCRT-III drives the formation of the intralumenal vesicles (ILVs) that capture the receptor within the MVB (Henne et al., 2012; Wollert and Hurley, 2010).

Although the ESCRT pathway was first identified in budding yeast in the context of MVB sorting of ubiquitylated cargoes, it supports several other unrelated cellular processes found in fission yeast, archaea and metazoans (Hanson and Cashikar, 2012; Peel et al., 2011). These include mitotic spindle maintenance (Frost et al., 2012; Morita et al., 2010), cytokinesis (Caballe and Martin-Serrano, 2011; Carlton and Martin-Serrano, 2007; Morita et al., 2007b) and viral budding (Garrus et al., 2001; Martin-Serrano and Neil, 2011). In addition, some MVB cargoes are sorted in ESCRT-dependent but ubiquitin-independent pathway(s) (Dores et al., 2012). These functionally distinct ESCRT pathways each have a unique complement of ESCRT complexes and accessory proteins associated with them (Bajorek et al., 2009; Carlton et al., 2008; Carlton et al., 2012; Morita et al., 2010; Peel et al., 2011). Hence, a crucial goal towards understanding the mechanism of EGFR downregulation is to identify the ESCRT pathway components that act selectively in this process.

**Fig. 1.**
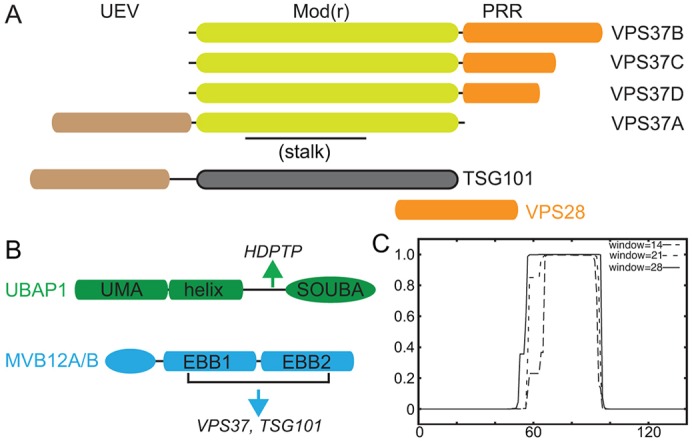
**Domain organisation and conservation of VPS37 and MVB12 families.** (A) Domain organisation of VPS37 family members. (B) Structural organisation of UBAP1- and MVB12-specific ESCRT-I complexes. (C) Coiled-coil prediction of the first 140 residues of UBAP1.

The ESCRT-I complex is central to all ESCRT pathways so far characterised. Yeast ESCRT-I is a heterotetramer, comprising vacuolar protein sorting protein 23, (Vps23p), Vps28p, Vps37p and MVB protein of 12 kDa (Mvb12p) (Katzmann et al., 2001; Kostelansky et al., 2007; Martin-Serrano and Neil, 2011). A core complex assembles from Vps23p, Vps28p and Vps37p, comprising a stalk region that involves helices from Vps23p and Vps37p, and a head region that includes part of Vps28p as well as Vps23p and Vps37p. Mvb12p associates primarily with the Vps23p–Vps37p stalk (Kostelansky et al., 2007). Mammals possess a single orthologue of Vps23p, tumor susceptibility gene 101 (TSG101) (Babst et al., 2000) and a single VPS28 (Bishop and Woodman, 2001), albeit with splice isoforms. Both proteins are evolutionarily conserved. In contrast, there are four VPS37-encoding genes expressed in mammalian cells (VPS37A–VPS37D) (Bache et al., 2004; Eastman et al., 2005; Stuchell et al., 2004) that resemble each other and Vps37p only within the ESCRT-I-core-forming domain, called the Mod(r) domain (Bache et al., 2004; Stuchell et al., 2004). Although the Mod(r) domains of VPS37B and VPS37C are highly similar (47% amino acid identity), those of VPS37A and VPS37D are more divergent (29% and 39% identity with VPS37B, respectively). VPS37A is particularly divergent in its stalk-forming region (22% identity compared with VPS37B versus 49% for VPS37C compared with VPS37B). VPS37A also has an N-terminal ubiquitin enzyme variant (UEV) domain, whereas VPS37B–VPS37D have C-terminal proline-rich regions of differing lengths (see Fig. 1) (Bache et al., 2004; Eastman et al., 2005; Stuchell et al., 2004).

There are three known mammalian proteins that serve as equivalents to Mvb12p based on their ability to integrate into ESCRT-I: MVB12A, MVB12B (Konishi et al., 2006; Morita et al., 2007a; Tsunematsu et al., 2010) and UBAP1 (Agromayor et al., 2012; Stefani et al., 2011). However, there is no sequence conservation between any of these proteins and Mvb12p. MVB12A and MVB12B each utilise conserved regions (EBB1 and EBB2) towards their C-termini to bind to the VPS37–TSG101 stalk (Morita et al., 2007a) (Fig. 1B). EBB2 resembles an N-terminal region within UBAP1, termed the UBAP1-MVB12-associated (UMA) domain (Agromayor et al., 2012; de Souza and Aravind, 2010; Stefani et al., 2011). Aside from this, UBAP1 is quite distinct from MVB12A and MVB12B. It contains a predicted coiled-coil-forming helix (residues 59–92) (Fig. 1C), a central region (residues 159–308), which binds the ESCRT accessory protein HD-PTP (Stefani et al., 2011), and a C-terminal arrangement of three overlapping UBA domains (residues 381–502) termed a solenoid of overlapping UBA domains (SOUBA) (Fig. 1B) (Agromayor et al., 2012; de Souza and Aravind, 2010; Stefani et al., 2011).

UBAP1 is important for the MVB sorting and degradation of ubiquitylated endosomal cargoes, including EGFR, but not for cytokinesis or retroviral budding (Agromayor et al., 2012; Stefani et al., 2011). By contrast, modulating the levels of MVB12A or MVB12B profoundly affects the assembly of infective retroviral particles (Morita et al., 2007a), whereas siRNA-mediated depletion of MVB12A or MVB12B only mildly affects the stability of EGFR, as assessed by western blotting (Tsunematsu et al., 2010). Previous studies from our laboratory have provided evidence that UBAP1 selectively associates with ESCRT-I complexes containing VPS37A, supporting a model of functionally distinct ESCRT-I types of defined MVB12 and VPS37 composition (Stefani et al., 2011). In contrast, however, several other studies based on expression of epitope-tagged components have found no such selectivity (Agromayor et al., 2012; Morita et al., 2007a; Tsunematsu et al., 2010), suggesting that the incorporation of VPS37 and MVB12 family members might be stochastic. To further test the selectivity of ESCRT-I function and assembly, we have compared the function of UBAP1 with that of MVB12A and MVB12B during ubiquitin-dependent EGFR sorting to the MVB. In conjunction, we have used cellular and *in vitro* systems in order to determine the selectivity of pairings of VPS37 and MVB12 family members within ESCRT-I. We demonstrate that the generation of at least a subset of ESCRT-I types is highly selective, matching their functional specialisation, and we have mapped the principal determinants that underlie this selectivity.

## RESULTS

### UBAP1, but not MVB12A or MVB12B, is crucial for ubiquitin-dependent EGFR sorting

**Fig. 2.**
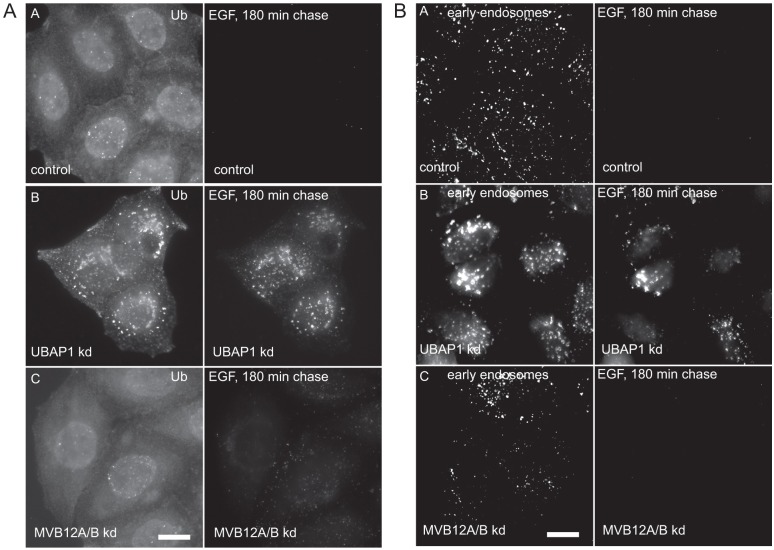
**UBAP1, but not MVB12A or MVB12B, regulates ubiquitin-dependent EGFR trafficking.** HeLaM cells were depleted of UBAP1 (UBAP1 kd), or of MVB12A and MVB12B (MVB12A/B kd). Cells were incubated with fluorescent EGF for 3 hours, then fixed and stained for ubiquitylated protein (A), or the early endosome marker EEA1 (B). Scale bars: 20 µm.

To test for specialisation in ESCRT-I function, we transfected cells with siRNA directed either against UBAP1 (Stefani et al., 2011) (supplementary material Fig. S1A), or against both MVB12A and MVB12B (supplementary material Fig. S1B), and directly compared the effects on EGFR trafficking. Note that because we failed to detect MVB12B in HeLaM cells, in line with previous reports that this protein has a restricted expression (Morita et al., 2007a), RNA interference was assessed by a failure to detect exogenously expressed protein. Cells were examined for transport of fluorescent EGF to lysosomes (assessed by loss of fluorescence signal), because disruption of this pathway, and a consequent build-up of fluorescent EGF in ubiquitin-rich early endosomes, is an excellent reporter of loss of ESCRT-I and UBAP1 function at the endosome (Doyotte et al., 2005; Stefani et al., 2011). As shown in Fig. 2A, depletion of UBAP1 arrested a pulse of fluorescent EGF in clustered compartments that labelled strongly for ubiquitylated proteins. Staining for early endosome antigen 1 (EEA1) confirmed that fluorescent EGF accumulated in clustered early endosomes in UBAP1-depleted cells (Fig. 2B), in agreement with an earlier report (Stefani et al., 2011). In contrast, cells depleted for MVB12A and MVB12B showed no apparent impairment in EGF degradation, as assessed by the loss of fluorescent EGF during a 3-hour chase. Images taken after a 30-minute chase confirmed that these cells internalised fluorescent EGF as well as control cells did (supplementary material Fig. S1C). In addition, the distribution of early endosomes and ubiquitylated proteins was similar in cells depleted of MVB12A and MVB12B to that in control cells. Depletion of UBAP1, but not MVB12A and MVB12B, caused the swelling of lysosomes (supplementary material Fig. S1D). In summary, these data indicate that UBAP1, and MVB12A and MVB12B are functionally distinct.

### The assembly of ESCRT-I complexes containing either UBAP1 or MVB12 is highly selective

**Fig. 3.**
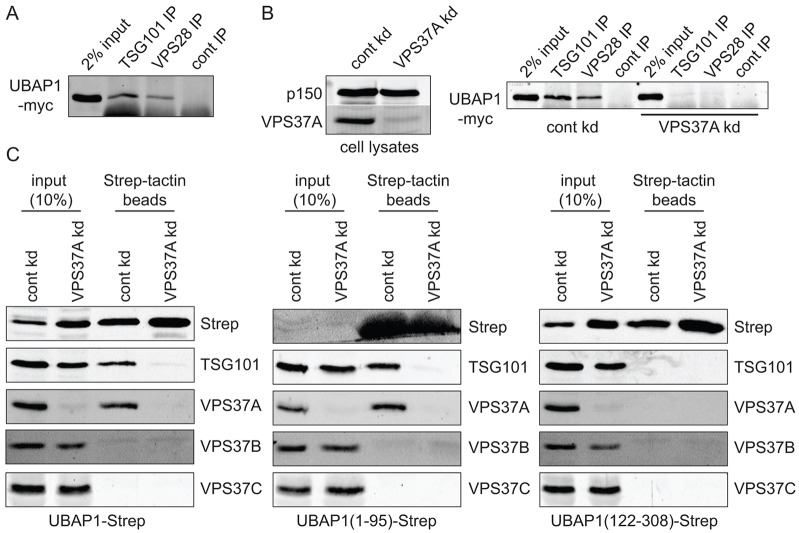
**Exogenously expressed UBAP1 selectively incorporates into VPS37A-containing ESCRT-I.** (A) UBAP1–Myc was transiently expressed and cell lysates immunoprecipitated (IP) with anti-TSG101 or anti-VPS28 antibodies. (B) Left panel: cells were siRNA-depleted for control (cont kd) or VPS37A (VPS37A kd) and western blotted for VPS37A and dynactin p150, as a loading control. Right panel: UBAP1–Myc was expressed in control- or VPS37A-depleted cells, and lysates were immunoprecipitated with anti-TSG101 or anti-VPS28 antibodies. (C) Full-length UBAP1–strep, or the UBAP1–strep N-terminal (1–95) or central (122–308) fragments, were transiently expressed in control- or VPS37A-depleted cells. Cell lysates were applied to Strep-Tactin beads, and samples were western blotted as indicated.

Our previous work, focussed on UBAP1 and based largely on native biochemistry, led us to conclude that MVB12 and VPS37 family members might combine selectively to assemble discrete ESCRT-I complexes (Stefani et al., 2011). Because these conclusions differ from those of other studies (Agromayor et al., 2012; Morita et al., 2007a; Tsunematsu et al., 2010), it was important to re-examine the subunit composition of ESCRT-I complexes in further detail. An inherent problem with co-expression studies is that all four ESCRT-I subunits are expressed at non-physiological levels, which might favour the efficient assembly of complexes that are otherwise rare. To minimise this risk, we transfected cells with one ESCRT-I subunit only and examined how this associated with native partners within ESCRT-I. As demonstrated previously (Stefani et al., 2011), epitope-tagged UBAP1 was efficiently incorporated into ESCRT-I, as judged by its ability to be immunoprecipitated with antibodies against TSG101 or VPS28 (Fig. 3A). Prior depletion of VPS37A alone completely abolished the ability of UBAP1–Myc to interact with TSG101 or VPS28 (Fig. 3B). Furthermore, upon expression of UBAP1–strep, pull-down using Strep-tactin beads recovered UBAP1–strep, TSG101 and VPS37A, but neither VPS37B nor VPS37C (Fig. 3C, left panel). The recovery of TSG101 with UBAP1–strep was completely abolished by depletion of VPS37A, even though total levels of endogenous TSG101, VPS37B and VPS37C were unaffected (Fig. 3C, left panel). Hence, even when VPS37A is absent, UBAP1 cannot utilise endogenous alternative VPS37 family members to incorporate into ESCRT-I. UBAP1–GFP, which is expressed at high levels, was also selectively incorporated into ESCRT-I-containing VPS37A, although a trace amount (over background levels) of VPS37B was also detected in UBAP1–GFP pull-downs (supplementary material Fig. S2). We could not find reagents against VPS37D that detected the endogenous protein.

Incorporation of UBAP1 into ESCRT-I requires the UMA domain (amino acids 17–63) (Agromayor et al., 2012; de Souza and Aravind, 2010; Stefani et al., 2011). Although we failed to express UBAP1(1-63)–strep (data not shown) a slightly longer N-terminal fragment, UBAP1(1-95)–strep, that incorporates an additional predicted α-helix (Fig. 2B), could be generated. Like full-length UBAP1–strep, this fragment was efficiently incorporated into ESCRT-I containing VPS37A, but not into ESCRT-I containing VPS37B or VPS37C. In addition, association of this fragment with TSG101 was absolutely dependent on the presence of VPS37A (Fig. 3C, centre panel). In contrast, a central fragment of UBAP1 known to associate with HD-PTP (Stefani et al., 2011), UBAP1(122-308)–strep, did not bind either TSG101 or VPS37A (Fig. 3C, right panel). Hence, we identified a minimal region of UBAP1 that retains the ability to selectively incorporate into an ESCRT-I complex within cells.

**Fig. 4.**
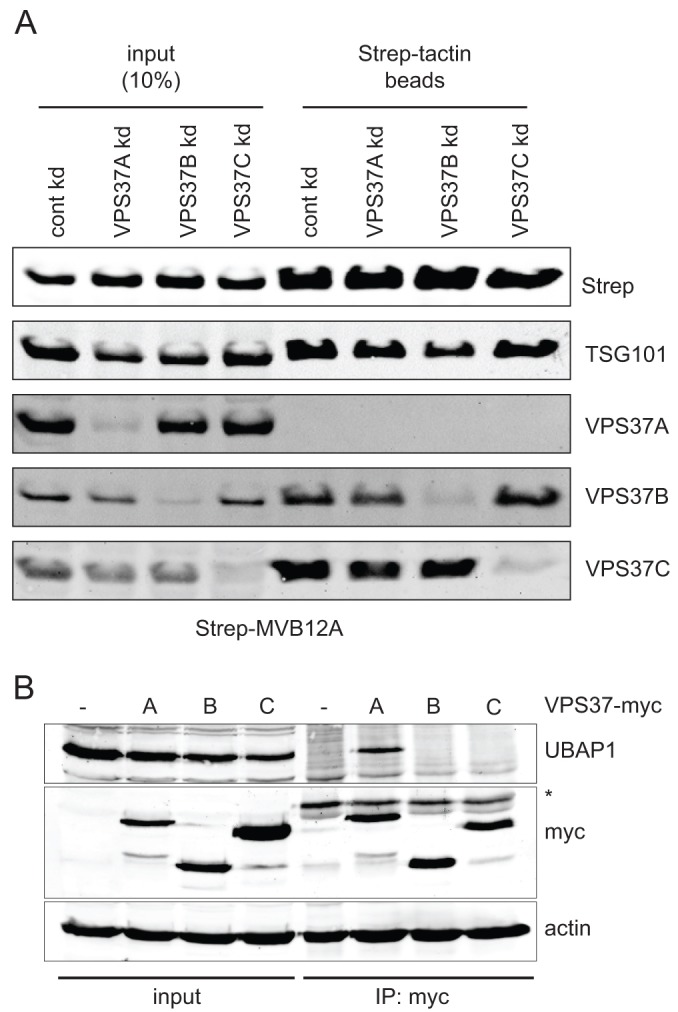
**MVB12A and VPS37C form a selective ESCRT-I complex.** (A) Strep-MVB12A was transiently transfected in control cells (cont kd) or cells depleted of VPS37A, VPS37B or VPS37C (VPS37A kd, VPS37B kd and VPS37C kd, respectively). Cell lysates were applied to Strep-Tactin beads, and samples were western blotted as indicated. (B) Lysates made from control cells (-) and cells expressing VPS37A–Myc, VPS37B–Myc or VPS37C–Myc (A, B and C, respectively) were immunoprecipitated with anti-Myc antibody and samples were western blotted as indicated. The asterisk indicates a non-specific band.

We next examined whether MVB12 incorporation into ESCRT-I was also selective, concentrating on MVB12A, the isoform expressed in HeLaM cells. MVB12A–strep was incorporated into ESCRT-I, shown by its ability to pull down endogenous TSG101 (Fig. 4A). It associated equally well with complexes containing VPS37B and VPS37C, but was not incorporated into ESCRT-I complexes containing VPS37A. Consistent with this, depletion of VPS37A did not influence the efficiency of MVB12A–ESCRT-I assembly (Fig. 4A). Hence, in contrast to other studies (Morita et al., 2007a; Tsunematsu et al., 2010), we find that although MVB12 shows no strong preference between VPS37B and VPS37C, it does not readily associate with VPS37A in cells. We then asked whether exogenously expressed VPS37 family members were also selectively incorporated into native ESCRT-I complexes. As shown in Fig. 4B, VPS37A–Myc, but neither VPS37B–Myc nor VPS37C–Myc, efficiently co-immunoprecipitated endogenous UBAP1.

### Selective assembly of ESCRT-I complexes *in vitro*

**Fig. 5.**
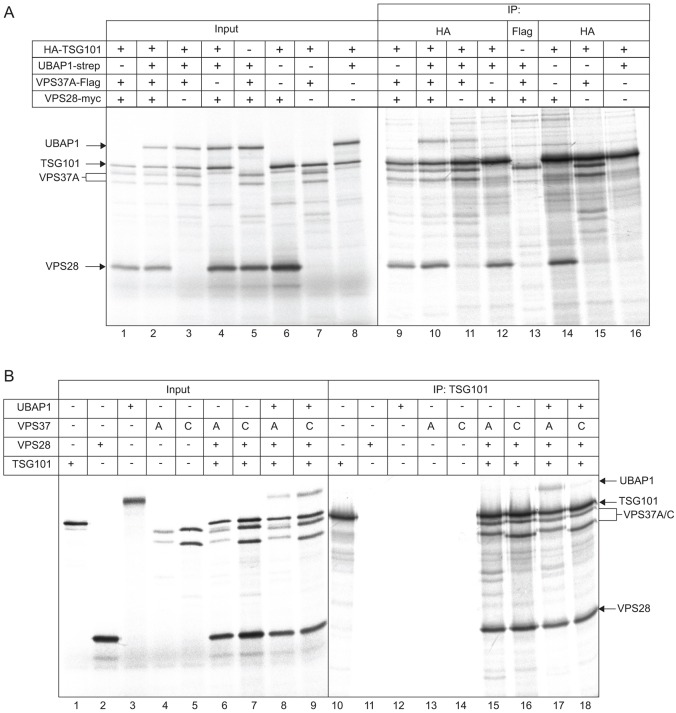
**Assembly of ESCRT-I complexes *in vitro*.** (A) The indicated combinations of UBAP1–strep, Flag–VPS37A, VPS28–Myc and HA–TSG101 were translated in rabbit reticulocyte lysates (left), and samples were immunoprecipitated (IP) as shown (right). (B) *In vitro* translation mixtures as indicated (left) were immunoprecipitated with anti-HA beads (right).

To further validate our findings, we expressed ESCRT-I components *in vitro*. Although human ESCRT-I can assemble when co-expressed in bacteria (Agromayor et al., 2012), we decided to generate recombinant proteins by translation in rabbit reticulocyte lysates. This system has the advantages that eukaryotic folding factors are present during protein synthesis and that labelled proteins are generated at nanomolar levels (Jackson and Hunt, 1983). Hence, ESCRT-I assembly conditions should more closely mirror those within the cell. We first found conditions that allowed UBAP1–strep, Flag–VPS37A, VPS28–Myc and HA–TSG101 to be co-translated in roughly equal amounts (Fig. 5A, left panel, lane 2). With the exception of a distinct doublet for VPS37A, the translation mixes were generally free of proteolytic fragments and/or incomplete translation products that might complicate analysis, although minor products were inevitably present. ESCRT-I was efficiently co-assembled, as judged by the ability to pull down each component on anti-HA beads (Fig. 5A, lane 10). As a control, no other components associated with anti-HA beads in the absence of HA–TSG101 (supplementary material Fig. S3A).

ESCRT-I assembly was coordinated correctly, given that efficient association of VPS28–Myc or Flag–VPS37A with HA–TSG101 occurred in the absence of additional ESCRT-I components in the translation mix (Fig. 5A, lanes 14 and 15), whereas UBAP1 associated with anti-HA beads only when Flag–VPS37A was present (Fig. 5A, lanes 10 and 12). In addition, no association was seen between VPS37A and any other ESCRT-I components in the absence of translated TSG101 (Fig. 5A, lane 13; supplementary material Fig. S3B). These findings are fully consistent with previous studies examining the assembly pathway of mammalian ESCRT-I (Bache et al., 2004; Bishop and Woodman, 2001; Eastman et al., 2005; Morita et al., 2007a; Stuchell et al., 2004) in which VPS28 and VPS37 can associate independently with TSG101, and MVB12 binds to a TSG101–VPS37 dimer. In addition, the data show that ESCRT-I assembly in reticulocyte lysates occurs exclusively between translation products, with no evidence for the involvement of any free endogenous ESCRT-I components that might be present in the lysates. Binding of UBAP1 to HA beads still occurred when VPS28 was absent, although it was significantly reduced (33% as efficiently as controls, ±3.0% s.e.m., *n* = 5; see supplementary material Fig. S3C for a further example). Previous work has shown that MVB12A and MVB12B assembly into ESCRT-I does not require VPS28 (Morita et al., 2007a), which fits well with the crystal structure of yeast ESCRT-I (Kostelansky et al., 2007), in which minimal contacts between Vps28p and Mvb12p occur. The orientation of UBAP1 with respect to VPS28 within the complex might therefore be slightly different to that of MVB12A and MVB12B. Importantly, the selectivity of ESCRT-I assembly was largely maintained under these conditions (Fig. 5B, compare lanes 17 and 18). Although UBAP1 was incorporated efficiently into complexes co-assembled with VPS37A, only a small amount of UBAP1 bound to complexes co-assembled with VPS37C (7.0%±1.23% s.e.m. compared to with VPS37A; *n* = 4), despite VPS37C binding to TSG101 as efficiently as VPS37A (117%±30.0% s.e.m., *n* = 7).

A further difference between bacterial expression and *in vitro* translation in rabbit reticulocyte lysates is the potential presence in the latter of post-translational modifications that might enhance the selectivity of ESCRT-I assembly. Of particular note, both MVB12A and MVB12B are subject to serine/threonine (Morita et al., 2007a) and tyrosine (Tsunematsu et al., 2010) phosphorylation. Hence, to rule out protein phosphorylation as a major determinant of selective ESCRT-I assembly, *in vitro* translations were also performed using wheat germ lysates because plant protein kinase systems are highly divergent from those of mammals. Additionally, no obvious UBAP1 orthologue is present in plants, and VPS37A is only poorly conserved [the most highly conserved region, the Mod(r) domain, is only 53% similar between human and wheat]. Therefore, assembly of the human ESCRT-I in wheat germ lysates minimises the possibility that endogenous proteins in the lysate aid selective ESCRT-I assembly. Indeed, assembly of wheat germ lysate translation products into ESCRT-I was both efficient and selective with respect to the fate of UBAP1 (supplementary material Fig. S3D, lanes 17 and 18).

### Mapping the determinants of selective ESCRT-I assembly

**Fig. 6.**
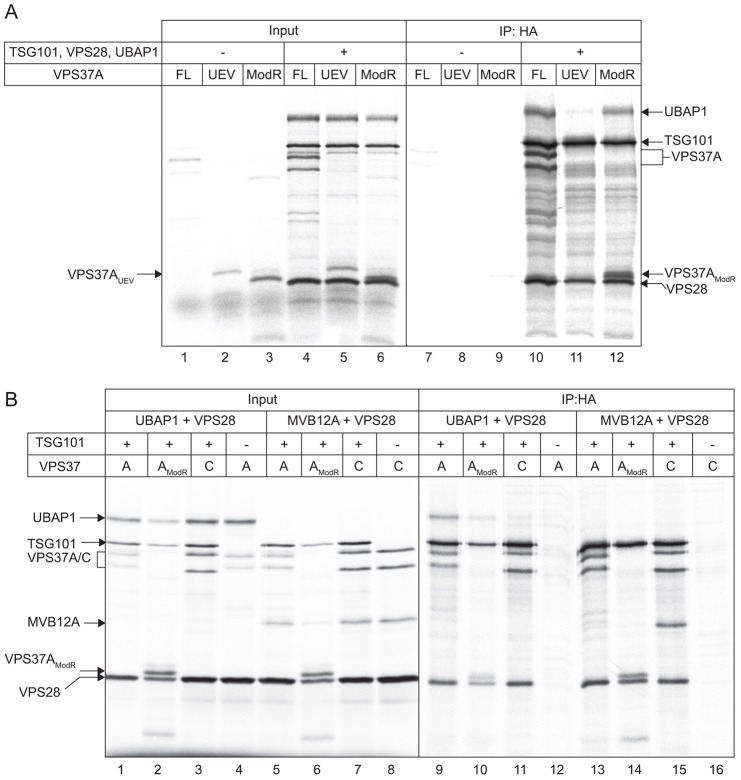
**The VPS37A Mod(r) domain confers selective assembly of UBAP1 into ESCRT-I.** (A) Domains of Flag–VPS37A were translated with or without other ESCRT-I components (left) and immunoprecipitated (IP) with anti-HA beads (right). FL, full length. (B) Translations performed as indicated, with or without MVB12A, (left) were immunoprecipitated with anti-HA beads (right). Note that MVB12A translations were consistently lower in the presence of the VPS37A(Mod(r)) domain.

Biochemical specificity in the binding of EBB and/or UMA domains to Mod(r)–TSG101 dimers could explain the selective ESCRT-I assembly we observe because these regions are known to drive assembly of MVB12A and MVB12B into ESCRT-I. These regions are divergent in UBAP1 and VPS37A compared to MVB12A and MVB12B, and VPS37B and VPS37C. We first tested which minimal region of VPS37A was required for selective incorporation of UBAP1 into ESCRT-I. Co-translations were performed using either full-length VPS37A, or either the UEV or Mod(r) domains. In contrast to full-length Flag–VPS37A, Flag–VPS37A(UEV) did not co-immunoprecipitate with HA–TSG101 from translation mixtures (Fig. 6A, compare lanes 10 and 11), despite a very weak association of the VPS37A UEV domain with TSG101 having been reported previously (Bache et al., 2004). As expected, almost no UBAP1 was co-immunoprecipitated from this mixture. Furthermore, no direct association between translated Flag–VPS37A(UEV) and UBAP1–strep could be detected (data not shown). Instead, UBAP1 co-immunoprecipitated with HA–TSG101 in the presence of Flag–VPS37A(Mod(r)) as efficiently as with full length Flag–VPS37A (Fig. 6A, compare lanes 10 and 12). The amount of VPS37A(Mod(r)) recovered in HA–TSG101 immunoprecipitates relative to full-length VPS37A was 77%±15% s.e.m. (*n* = 8), whereas the recovery of UBAP1 was 76%±5% (*n* = 6). In contrast, strep–MVB12A did not associate with complexes containing full-length Flag–VPS37A or Flag–VPS37A(Mod(r)), but associated strongly with those containing Flag–VPS37C (Fig. 6B). Hence, as described for MVB12A and MVB12B (Morita et al., 2007a), UBAP1 associates with a Mod(r)-domain–TSG101 dimer, but in this case only those containing the VPS37A Mod(r). Moreover, the Mod(r) domain of VPS37A does not readily bind MVB12A, consistent with the selective assembly of MVB12 into ESCRT-I that we observe in cells.

**Fig. 7.**
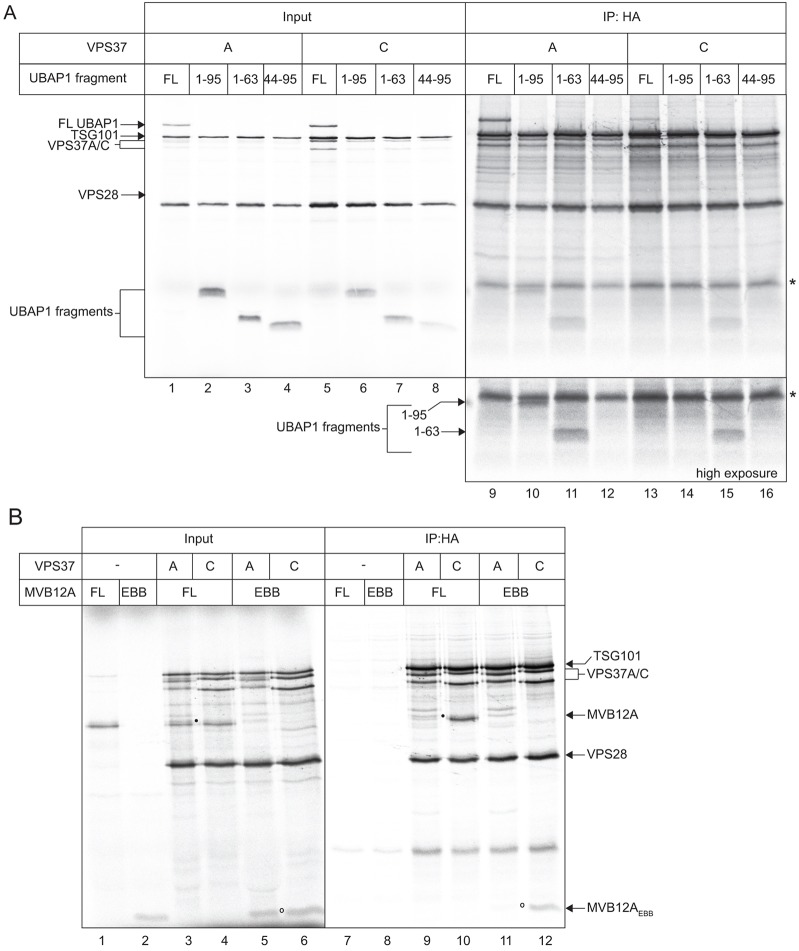
**Mapping the regions of UBAP1 and MVB12A that confer selective ESCRT-I assembly.** (A) *In vitro* translations containing the indicated fragments of UBAP1 and epitope-tagged ESCRT-I subunits (left) were immunoprecipitated (IP) with anti-HA (TSG101) beads (right). The asterisks denote a background band (probably label bound to globin). A section of the image is shown at higher exposure to highlight the behaviour of the short UBAP1 fragments, which each contain a single methionine residue. An independent experiment is shown in supplementary material Fig. S4. FL, full length. (B) Translations as indicated (left), with full-length MVB12A (filled circle) or the EBB1–EBB2 fragment (open circle), were immunoprecipitated with anti-HA beads (right).

**Fig. 8.**
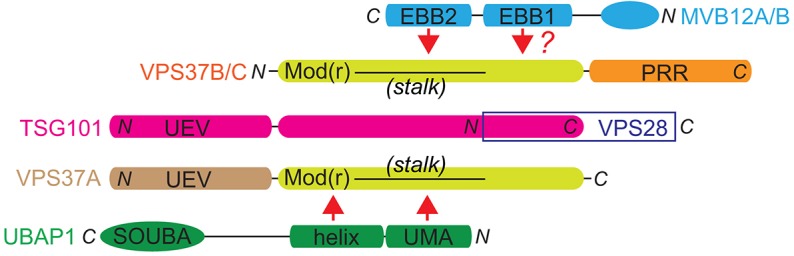
**A summary of selective ESCRT-I assembly.** The TSG101 core can recruit VPS37B and/or VPS37C in combination with MVB12A or MVB12B (upper interaction), or VPS37A in combination with UBAP1 (lower interaction). Integration of UBAP1 or MVB12A or MVB12B requires recognition of the UMA and EBB2 domains by corresponding Mod(r) domains in VPS37, probably in the stalk regions, but additional interactions involving the UBAP1 helix (and perhaps the EBB1 region of MVB12) confer specificity of assembly.

Next, the UMA domain of UBAP1 (amino acids 1–63), and the EBB1–EBB2 region of MVB12A were co-translated with other ESCRT-I components. UBAP1(UMA) incorporated into ESCRT-I efficiently relative to full-length UBAP1 (Fig. 7A). Hence, the minimal information necessary for assembly of UBAP1 with TSG101 is UBAP1(1-63) and VPS37(Mod(r)) (supplementary material Fig. S4A). Strikingly, however, the selectivity of this association was almost completely lost, because UBAP1(UMA) also associated readily with VPS37C-containing ESCRT-I (Fig. 7A, compare lanes 11 and 15). In contrast, a fragment of UBAP1 containing the UMA domain and the neighbouring predicted helical region (amino acids 1–95) was incorporated into ESCRT-I as selectively as the full-length protein (Fig. 7A, compare lanes 10 and 14). No binding could be detected between the UBAP1 predicted helix region alone (amino acids 45–95) and ESCRT-I (Fig. 7A, lane 12). Note that the closeness of a non-specific band to the UBAP1(1-95) fragment prevented reliable quantification (an independent experiment is shown in supplementary material Fig. S4B). Hence, the UMA domain might fully account for the efficiency of UBAP1 incorporation into ESCRT-I, but the neighbouring region helps restrict its association to ESCRT-I containing VPS37A. MVB12A(EBB1-EBB2) bound VPS37C-containing ESCRT-I efficiently compared with full-length MVB12A, but did not associate with VPS37A-containing ESCRT-I (Fig. 7B, compare lanes 11 and 12). Hence, this region of MVB12A is sufficient to confer selective ESCRT-I binding. In summary, we have identified peptide regions within MVB12 and VPS37 family members that confer their selective assembly into functionally distinct ESCRT-I complexes (Fig. 8).

## DISCUSSION

Our studies reveal that selective association between MVB12 and VPS37 subunit family members generates distinct ESCRT-I complexes that correlate with different biological activities. Hence, although stochastic association of known MVB12 and VPS37 family members would generate up to 12 different ESCRT-I complexes, our studies suggest that the cellular ESCRT-I complement is rather more restricted. Discrimination between UMA and Mod(r) domains contributes to the selectivity of ESCRT-I assembly, although ‘unmatched’ UMA and Mod(r) domain pairings might bind each other with low affinity and hence support less-selective assembly of ESCRT-I when multiple subunits are co-expressed. The Mod(r) domains of all VPS37 family members are predicted to form similar, largely helical, structures, though it is noteworthy that the level of conservation between VPS37A and other family members diminishes within the stalk-forming region of the Mod(r) domains compared to the head-forming region. Based on the structure of yeast ESCRT-I (Kostelansky et al., 2007), it is the stalk-forming region that is likely to form the principal interface with MVB12. We provide evidence that other domains might regulate the binding reactions. Specifically, a putative helix contiguous with the UMA domain of UBAP1 appears important for preventing the non-selective association of UBAP1 with VPS37C. Although this region might also enhance the avidity of UBAP1 for VPS37A, we found no direct evidence of this. The EBB1 and EBB2(UMA) regions of MVB12A and MVB12B each contain sufficient information to bind ESCRT-I (Morita et al., 2007a) and, in combination, are likely to form a binding surface that is more selective than the EBB2(UMA) sequence alone, although we have not tested this possibility.

Although the incorporation of UBAP1 into ESCRT-I appears to be highly selective, other ESCRT-I complexes might well form in a more stochastic fashion. For example, the lack of preference of exogenous MVB12A for integrating into ESCRT-I complexes containing native VPS37B or VPS37C is in keeping with the greater similarity in sequence and domain organisation of these VPS37 family members. Hence, MVB12A and MVB12B, and VPS37B and VPS37C, might integrate into ESCRT-I interchangeably, and perhaps function in related processes. We have not examined the potential for known MVB12 proteins to associate with VPS37D, which is less well characterised. Additionally, there might be further MVB12 family members that await identification. It is also noteworthy that our previous study suggested that a minor portion of VPS37A might lie outside of an UBAP1-containing ESCRT-I (Stefani et al., 2011). Clearly, further work is required to fully characterise the range of cellular ESCRT-I complexes.

The insensitivity of ubiquitin-dependent EGFR sorting to the combined depletion of MVB12A and MVB12B is consistent with the distinct ESCRT-I assembly pathway that we report here. However, it remains quite possible that MVB12A and MVB12B have some role at the endosome, and/or in event(s) downstream of EGFR distinct from UBAP1. Both MVB12A and MVB12B associate with EGFR, and their patterns of tyrosine phosphorylation alter upon EGFR activation (Tsunematsu et al., 2010). Also consistent with an endosomal function, MVB12A binds CD2AP and CIN85, known regulators of EGFR signalling and endosomal trafficking (Konishi et al., 2006). Furthermore, loss of TSG101 causes more severe defects in endosome morphology than loss of UBAP1 (Doyotte et al., 2005; Stefani et al., 2011). This might point to the presence of additional ESCRT-I complexes acting alongside that containing UBAP1, though it is also possible that a TSG101–VPS28–VPS37A complex lacking UBAP1 retains some sorting activity. The documented interaction between VPS37C and the ESCRT-0 component, hepatocyte growth factor receptor substrate (Hrs) (Eastman et al., 2005), is also consistent with the possibility that other ESCRT-I complexes have some endosomal function. By analogy, ESCRTs perform two roles during cytokinesis; they drive membrane scission but also promote microtubule disassembly by recruiting the microtubule severing protein, spastin (Caballe and Martin-Serrano, 2011; Yang et al., 2008).

The finding that ubiquitin-dependent EGFR sorting requires an ESCRT-I complex of unique composition extends the conclusions of several studies identifying ESCRT components or ESCRT-interacting proteins that are involved only in certain cellular processes. For example, although ESCRT-0 initiates the pathway at the endosome (Hurley and Stenmark, 2011; Komada and Soriano, 1999; Raiborg and Stenmark, 2009; Raiborg et al., 2002; Razi and Futter, 2006; Ren and Hurley, 2010; Wollert and Hurley, 2010), the centrosomal protein CEP55 and the p6 late domain of Gag perform this role during cytokinesis and HIV budding, respectively (Caballe and Martin-Serrano, 2011; Carlton and Martin-Serrano, 2007; Morita et al., 2007b; Peel et al., 2011; von Schwedler et al., 2003; Strack et al., 2003). These interactions would negate the need for ESCRT-0 in these events, although interestingly, binding does occur between CEP55 and ESCRT-0 (Morita et al., 2007b). Bro1 proteins support ESCRT function in various cellular contexts and, like ESCRTs, are divergent in higher eukaryotes. One Bro1 protein, Alix promotes CHMP4B recruitment during cytokinesis and viral budding (Caballe and Martin-Serrano, 2011; Fisher et al., 2007), and is required for sorting of some endocytic cargo (Baietti et al., 2012; Dores et al., 2012; Hanson and Cashikar, 2012; Matsuo et al., 2004; Peel et al., 2011). Meanwhile, another Bro1 protein, HD-PTP, plays a part in CHMP4 recruitment during EGFR sorting (Ali et al., 2013). The selective role that HD-PTP appears to have over Alix in ubiquitylated MVB cargo sorting (Bowers et al., 2006; Cabezas et al., 2005; Doyotte et al., 2008) is underlined by its selective binding to the ESCRT-0 subunit signal transducing adaptor molecule (STAM), and to UBAP1 (Ali et al., 2013; Stefani et al., 2011). A further ESCRT component with selective function is IST1, an ESCRT-III subunit that is essential for cytokinesis but not for EGFR sorting or virus budding (Agromayor et al., 2009; Bajorek et al., 2009). IST1 binds a further effector, MITD1, which also acts selectively during cytokinesis (Hadders et al., 2012; Lee et al., 2012). Hence, our understanding of the divergence between ESCRT pathways continues to expand. This study elucidates the molecular basis for one such specialisation.

## MATERIALS AND METHODS

### Reagents

Antibodies against the following proteins were used. Mouse: EEA1 (Transduction Labs, Oxford, UK); LAMP1 (Developmental Studies Hybridoma Bank, University of Iowa); UBAP1 (Abnova Corporation); ubiquitin-protein conjugates (FK2; Enzo Life Sciences, Exeter, UK); TSG101, tubulin and actin (AbCam; Cambridge, UK); Myc (Millipore, UK); strep (IBA, Göttingen, Germany); HA (Sigma, Poole, UK), MVB12A (Abcam); Flag (Sigma); dynactin subunit 1 (p150; Transduction Labs). Rabbit: VPS37A and UBAP1 (Proteintech Europe, Manchester, UK); TSG101 and VPS28 (house generated; Stefani et al., 2011). Fluorescent secondary antibodies were from Jackson ImmunoResearch Laboratories (PA, USA). IRDye 680 CW and IRDye 800 CW secondary antibodies for western blotting were from Li-Cor Bioscience (Cambridge, UK).

### DNA

MVB12B and VPS37B with C-terminal Myc-Flag tags in pCMV6-Entry were purchased from Origene (Rockville, MD, USA). VPS37A–Myc and VPS37C–Myc were in pcDNA5. For *in vitro* translations, the following constructs were used: HA–TSG101 in pGADT7, full-length and the Mod(r) domain (amino acids 229–397) of VPS37A, and VPS37C were in pcDNA3.1^+^ containing an N-terminal Flag tag. VPS28–Myc was in pcDNA3.1^+^ containing a C-terminal Myc tag. Full-length MVB12A, and fragments 1–63, 1–95, 44–95 and 122–308 of UBAP1 were in pTriEX-5 with C-terminal strep tags. The coiled coil prediction for UBAP1 was performed by COILS (http://www.ch.embnet.org/software/COILS_form.html) (Lupas et al., 1991).

### Cell culture

For siRNA experiments, 20 nM of each siRNA duplex was transfected using INTERFERin (Polyplus; Illkirch, France), as specified by the manufacturer, and samples were harvested after 72 hours. To confirm the knockdown of MVB12A and MVB12B, cells were also transiently transfected with both DNA constructs after 48 hours. Sequences for the UBAP1 duplex are as described previously (Stefani et al., 2011), and for MVB12A+B knockdowns, SMARTpools containing four duplexes were purchased from Thermo Fisher (catalogue numbers L-026207 and L-015563; Loughborough, UK). AllStars negative control siRNA was from Qiagen (Manchester, UK). For uptake of Alexa-Fluor-488-conjugated EGF (Invitrogen), cells were grown on glass coverslips and incubated with labelled EGF for 5 minutes at 37°C in binding medium [L-15 medium (Invitrogen) containing 0.2% (w/v) BSA], then chased in binding medium containing unlabelled EGF before fixation. Times refer to total pulse-chase times. For MG132 treatments cells were incubated with 1 µM for 18 hours.

### Immunofluorescence and imaging

For immunofluorescence, cells were fixed in 3% formaldehyde and quenched with glycine and permeabilised for 3 minutes in 0.1% (w/v) Triton X-100. For LAMP staining, cells were fixed in methanol at −20°C. DNA was stained using 4′,6-diamidino-2-phenylindole (DAPI). Fluorescence was examined using a wide-field Olympus BX-60 microscope fitted with a 60× 1.40 N.A. PlanApo objective and a CoolSnap ES camera (Roper Scientific, Ottobrunn, Germany), with images captured using MetaVue. Images were opened as 16-bit grey-scale images and scaled using linear transformations in ImageJ, then converted to 24-bit RGB files in PhotoShop CS before being placed in Adobe Illustrator. For EGF trafficking experiments, all samples were imaged and adjusted for image contrast using identical settings.

### Co-immunoprecipitation

At 24 hours post-transfection cells were lysed in IP buffer (10 mM Tris-HCl pH 7.4, 150 mM NaCl, 1 mM EDTA) supplemented with protease inhibitor cocktail, 1 mM PMSF and 0.5% CHAPS. After incubation at 4°C for 1 hour, insoluble material was removed by centrifugation at 14,000 ***g*** for 10 minutes. A sample of the total cell lysate (input) was retained for analysis, and immunoprecipitations were performed at 4°C for 18 hours, before the resin was washed with IP buffer and SDS-PAGE sample buffer was added. UBAP–strep was immunoprecipitated using Strep-Tactin beads (Thermo Fisher, UK), and UBAP–GFP was immunoprecipitated using GFP–TRAP (ChromoTek, Martinsred, Germany). Protein-A–Sepharose (Genscript, Piscataway, NJ, USA) with the appropriate antibody, was used for all other immunoprecipitations.

### *In vitro* translation

RNAs were synthesised from PCR products using T7 RNA polymerase (Promega, Southampton, UK). To generate the EBB fragment of MVB12A (amino acids 192–273) a T7 promoter sequence was included in the forward primer and the full-length DNA was used as a template. Proteins were translated in 15 µl reactions of nuclease-treated rabbit reticulocyte lysate (Promega) containing ^35^S-methionine (Perkin Elmer, Buckinghamshire, UK), and 100 units of RNasin (Promega) for 1 hour at 30°C. Samples were then incubated at 30°C with 1 mM puromycin for 10 minutes. Alternatively, wheat germ translations were incubated at 25°C, and an additional incubation with 0.5 mg/ml RNaseA for 5 minutes at 37°C was included post-translation, followed by centrifugation at 10,000 ***g*** for 5 minutes. A 10% sample of the input was retained for analysis, and IP buffer (10 mM Tris-HCl pH 7.4, 150 mM NaCl, 1 mM EDTA) supplemented with protease inhibitor cocktail, 1 mM PMSF and 0.1% Triton X-100 was used to dilute the remaining sample tenfold. A pre-clearing step was performed using 20 µl Protein-A–Sepharose (Genscript), 10 mM cold L-methionine, 1 mM PMSF and protease inhibitor cocktail for 1 hour at 4°C. For translations of small fragments, an additional pre-clearing step using Ni-NTA resin (Sigma) was also included to remove the haemoglobin. Samples were clarified by centrifugation at 14,000 ***g*** for 10 minutes at 4°C, and immunoprecipitations were either performed overnight with the indicated antibodies, and then Protein-A–sepharose for 2 hours the following day, or, for TSG101, were performed using anti-HA-conjugated agarose (Sigma) overnight. After washing three times in immunoprecipitation buffer, SDS-PAGE sample buffer was added to the resin for analysis. Following SDS-PAGE, the gels were dried and visualised by phosphoimaging using a BAS5000 imager (Raytest; Surrey, UK). AIDA software was used for quantification.

## Supplementary Material

Supplementary Material
