# Supplementary material for: The molecular basis for selective assembly of the UBAP1-containing endosome-specific ESCRT-I complex
Source: J Cell Sci. 2014 Feb 1;127(3):663–72. doi: 10.1242/jcs.140673 (PMC4007767; doi:10.1242/jcs.140673)
Supplement: Supplementary Material [file supp_127_3_663__index.html]

The molecular basis for selective assembly of the UBAP1-containing endosome-specific ESCRT-I complex — Supplementary Material 

# The molecular basis for selective assembly of the UBAP1-containing endosome-specific ESCRT-I complex

## JCS140673 Supplementary Material

**Files in this Data Supplement:**

- **Supplementary Material PDF**
