# Supplementary material for: The molecular basis for selective assembly of the UBAP1-containing endosome-specific ESCRT-I complex
Source: J Cell Sci. 2014 Feb 1;127(3):663–72. doi: 10.1242/jcs.140673 (PMC4007767; doi:10.1242/jcs.140673)

Supplementary Material

Supplementary Figure Legends

Figure S1

(**A**) HeLaM cells were treated with control or UBAP1 siRNA. Cell lysates were blotted for UBAP1, or for tubulin as a loading control. (**B**) HeLaM cells were treated with control siRNA or Smartpool mixtures for MVB12A and MVB12B. Cells were then transfected with strep-tagged MVB12A and myc-Flag-tagged MVB12B as indicated. Lysates were immunoblotted for MVB12A, MVB12B, or for tubulin as a loading control. Asterisks show non-specific bands. (**C**) HeLaM cells depleted of MVB12A and MVB12B were incubated with fluorescent EGF (green) for 30 min, then fixed and stained for LAMP1 (red). Scale bar = 20 μm. (**D**) HeLaM cells were depleted of UBAP1, or of MVB12A and MVB12B. Cells were incubated with fluorescent EGF for 3 hr, then fixed and stained for LAMP1. Scale bar = 20 μm.

Figure S2

UBAP1-GFP, or GFP as a control, were transiently expressed. Cell lysates were applied to GFP-Trap beads, and samples were Western blotted as indicated.

Figure S3

(**A**) ESCRT-I components were translated as indicated, and immunoprecipitated with anti-HA beads. (**B**) ESCRT-I subunits were translated as indicated (left) and then immunoprecipitated with the indicated anti-tag antibodies (right). (**C**) ESCRT-I subunits were translated with or without VPS28 and immunoprecipitated with anti-HA (TSG101). (**D**) Mixtures of ESCRT-I subunits were translated in wheat-germ lysates (left) and immunoprecipitated with anti-HA (right). The * indicates a non-specific band that most likely represents aminoacyl tRNAs.

Figure S4

(A, B) ESCRT-I components were translated as indicated (left), and immunoprecipitated with anti-HA beads (right). The * indicates a non-specific band.

Supplementary Figure 1

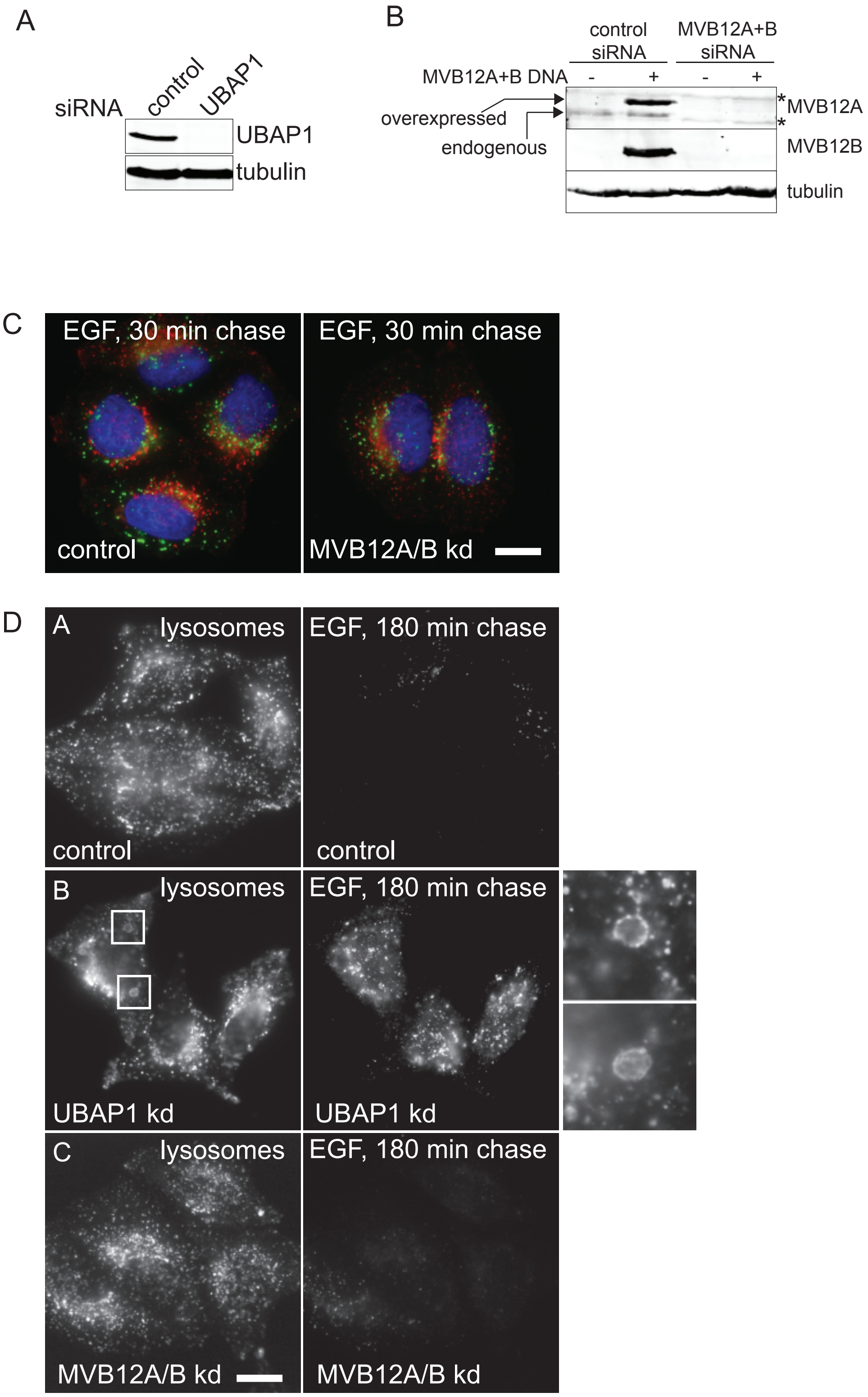

Supplementary Figure 2

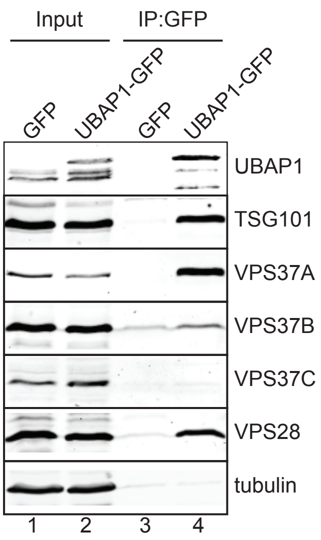

Supplementary Figure 3

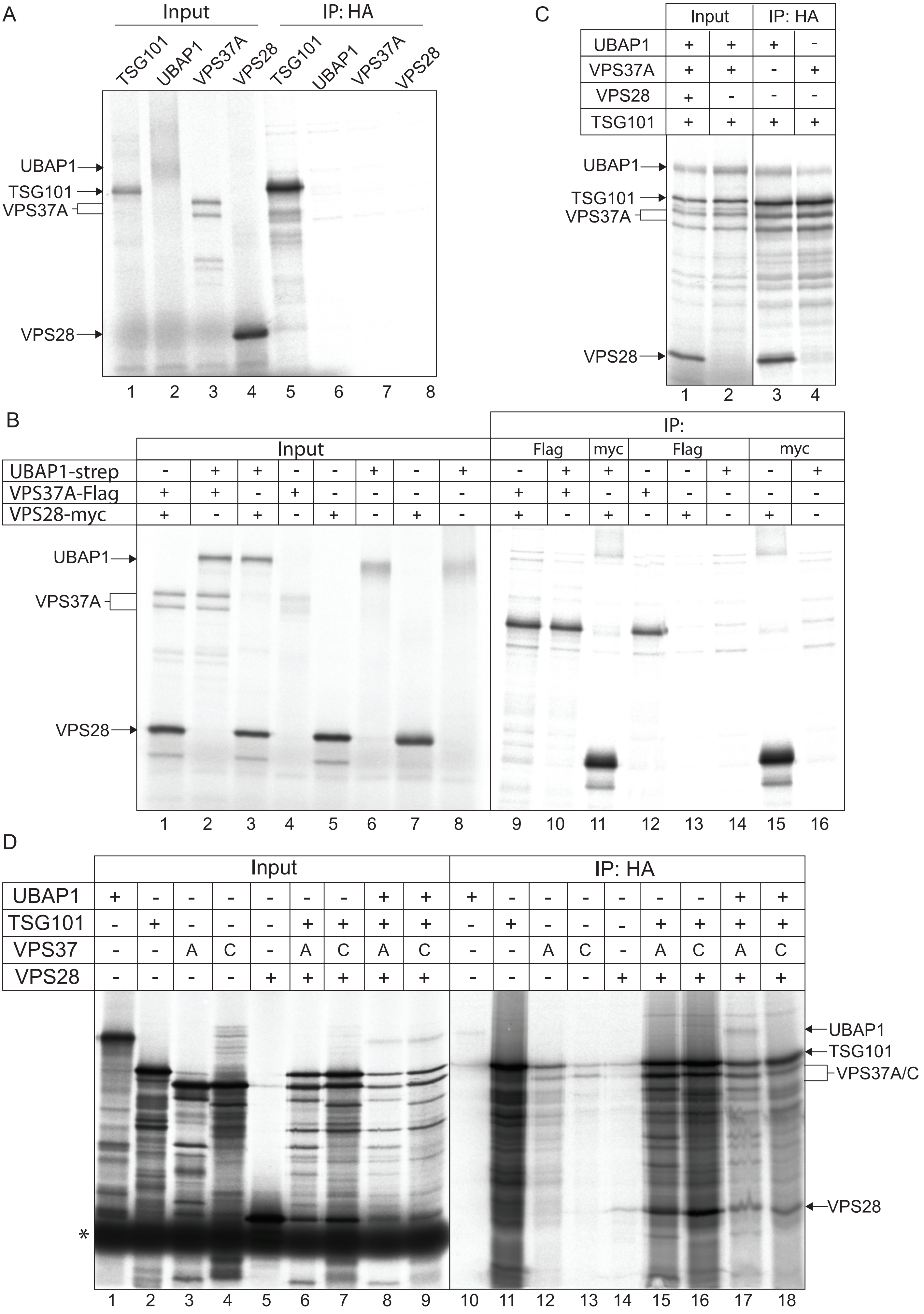

Supplementary Figure 4

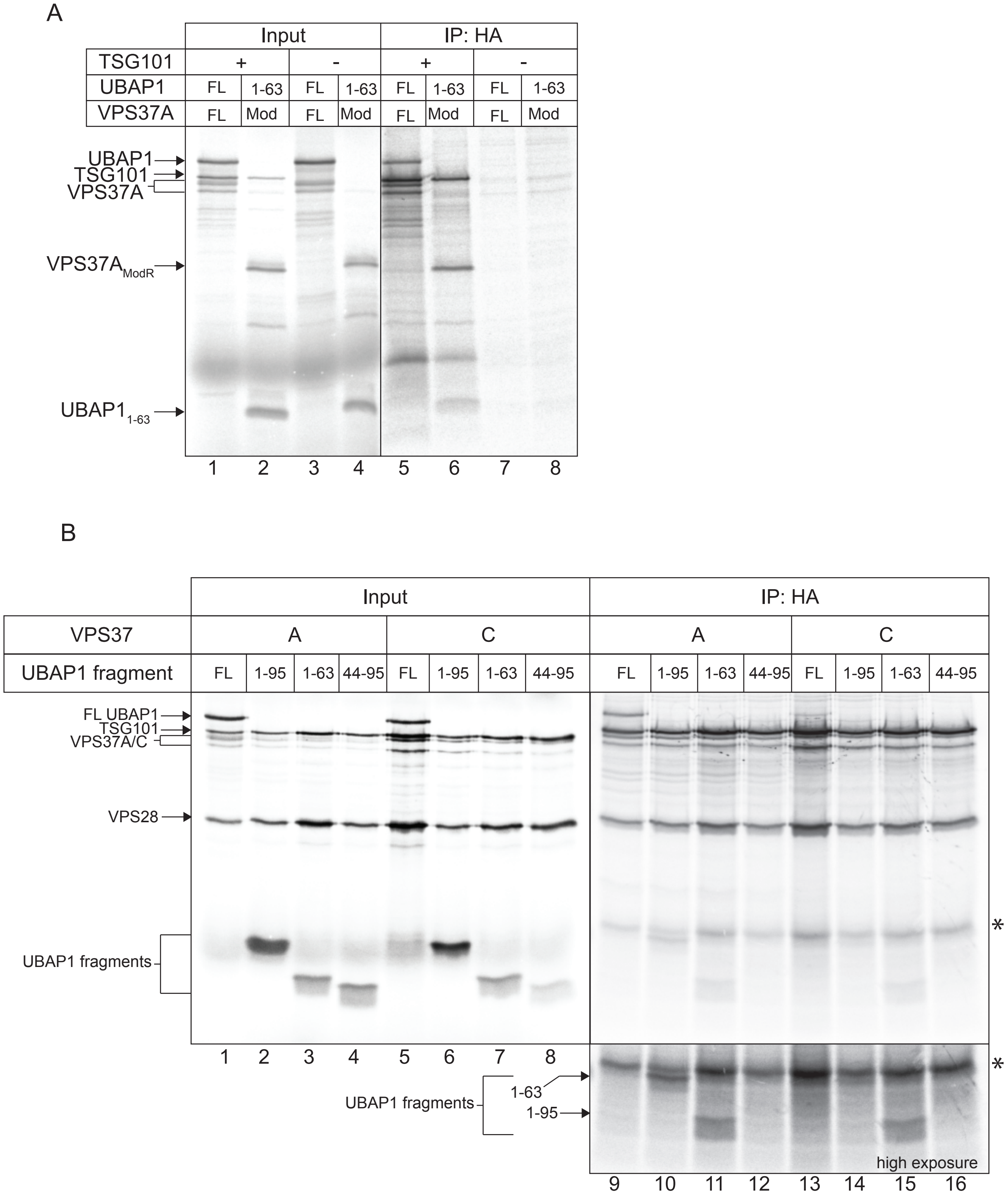

Supplement: Supplementary Material [file supp_127.3.663_JCS140673.pdf]
